# Clinical and Functional Characterization of Novel AGL Variants in Two Families with Glycogen Storage Disease Type III

**DOI:** 10.1155/2023/6679871

**Published:** 2023-05-30

**Authors:** Tingting Yu, Hao Fu, Aoyu Yang, Yan Liang

**Affiliations:** Department of Pediatrics, Tongji Hospital, Tongji Medical College, Huazhong University of Science and Technology, Wuhan, Hubei 430030, China

## Abstract

**Purpose:**

Glycogen storage disease type III (GSDIII) is a uncommon autosomal recessive inherited metabolic disorder, which is caused by variants in the AGL gene. The purpose of this study was to elucidate the clinical and functional features of two novel variants in two families with GSDIIIa.

**Methods:**

We collected the clinical and laboratory data of the two patients. Genetic testing was performed using GSDs gene panel sequencing, and the identified variants were classified according to the American College of Medical Genetics (ACMG) criteria. The pathogenicity of the novel variants was furthermore assessed through bioinformatics analysis and cellular functional validation experiments.

**Results:**

The two patients were hospitalized with abnormal liver function or hepatomegaly, which was characterized by remarkably elevated liver enzyme and muscle enzyme levels, as well as hepatomegaly, and were eventually diagnosed with GSDIIIa. Genetic analysis detected two novel variants of AGL gene in the two patients: c.1484A > G (p.Y495C), c.1981G > T (p.D661Y). Bioinformatics analysis indicated that the two novel missense mutations most likely altered the protein's conformation and therefore made the enzyme it encodes less active. Based on the ACMG criteria, both variants were considered likely pathogenic, in accordance with the functional analysis results, which demonstrated that the mutated protein was still localized in the cytoplasm and that the glycogen content of cells transfected with the mutated AGL was increased compared to cells transfected with the wild-type one.

**Conclusion:**

These findings indicated that the two newly identified variants in the AGL gene (c.1484A > G; c.1981G > T) were undoubtedly pathogenic mutations, inducing a slight reduction in glycogen debranching enzyme activity and a mild increase in intracellular glycogen content. Two patients who visited us with abnormal liver function, or hepatomegaly, improved dramatically after treatment with oral uncooked cornstarch, but the effects on skeletal muscle and myocardium required further observation.

## 1. Introduction

Glycogen storage disease type III (GSDIII) is an autosomal recessive inherited metabolic disease, which is caused by variants in the AGL gene leading to a deficiency in glycogen debranching enzyme (GDE) during the glycogen degradation pathway [1]. It is relatively rare with an estimated incidence of 1 : 100000 births [2]. GSDIII is a subtype of hepatic glycogen storage disease (GSDs), presenting with hepatomegaly, hepatic dysfunction, hypoglycemia, hyperlipidemia, growth retardation, as well as progressive myopathy and cardiomyopathy [3–8]. There are four subtypes in GSDIII classified based on the difference of the tissues involved and variated regions of the GSD enzyme. GSDIIIa is the most prevalent one which accounts for up to 80%, predominantly manifested as liver and muscle involvement. GSDIIIb, comprising approximately 15%, primarily affects the liver. The remaining two rare subtypes (GSDIIIc, GSDIIId) can be referable to a deficiency in the glucosidase and glucose-transferase activity, respectively [9]. The current diagnosis is mainly based on a combination of clinical features, enzymatic detection, and genetic testing [10]. Symptomatic treatments of high-fat diet, high-protein diet, and uncooked cornstarch supplementation are the main therapeutic approaches for GSDIII, but the treatment efficacy varies among individuals [11].

The AGL gene is located on chromosome 1p21 and contains 35 exons. It encodes a protein consisting of 1532 amino acids, which is mainly expressed in the liver and skeletal muscle [12]. GDE encoded by the AGL gene is a large monomer protein with two independent catalytic activities domain and a glycogen binding domain occurring at separate sites on a single polypeptide chain 1 [13]. It participates in the process of glycogen degradation, to maintain the balance of body energy metabolism. To date, a great many of variations of GSDIII have been defected, encompassing missense, duplication, deletions, frameshift, and splicing site. Notably, the splicing site variant (c.1735 + 1G > T) is the most prevalent mutation identified across all populations [14].

In this study, we reported two unrelated patients with GSDIII and identified four variants in the AGL gene. Moreover, cellular experiments would be conducted to further analyze the pathogenic consequence of the two novel missense variants.

## 2. Materials and Methods

### 2.1. Patients Enrollment and Ethical Approval

We investigated two children with GSDIII, admitted to Tongji Hospital, Tongji Medical College, Huazhong University of Science and Technology. Medical history and clinical data were collected, including age of disease onset, age of diagnosis, disease course, initial symptoms, serum biochemical parameters, and pathology of liver biopsy. The study was approved by the Ethics Committee of Tongji Hospital, Tongji Medical College, Huazhong University of Science and Technology (Approval number: TJIRB20180703).

### 2.2. Next Generation Sequencing and Bioinformatic Analysis

2 ml of peripheral venous blood samples was collected from the probands and their family members. Genomic DNAs were extracted by QIAamp Whole Blood DNA Extraction Kit (Qiagen, Germany). The GenCap liquid phase target genes capture technology was performed to capture the exon regions of GSD-related genes. About 20 GSD-related genes recorded in OMIM were included in this panel designed based on MyGenostics GenCap: AGL, ALDOA, EN03, G6PC, GAA, GSY1, GSY2, GBE1, GYG1, LDHA, PFKM, PGM1, PGAM2, PHKA1, PHKA2, PHKB, PHKG2, PYGL, PYGM, and SLC37A4. Then, the Illumina HiSeq *X* ten system was used for sequencing.

Genetic variants and their segregation in the family were confirmed by Sanger sequencing. Five kinds of bioinformatics software (Mutation Taster, Polyphen⁃2, SIFT, Provean, and PyMol) were performed to predict the damaging effect of the identified variants on AGL function, and PhyloP and GERP were used to analyze the conserved protein sequences of genes encoded by different species. The pathogenicity of genetic variations was evaluated according to the classification standard of genetic variation of ACMG.

### 2.3. Plasmid Construction and Cellular Transfection

The human wild-type *AGL* (NM_000642.3) and mutated AGL (c.1484A > G; c.1981G > T) full-length cDNAs were constructed into the pcDNA3.1-3xFlag vectors by Vigene Biosciences (Shandong, China). The sequences of wild-type and mutant AGL plasmids were confirmed by DNA sequencing. Human embryonic kidney 293T (HEK-293T) cells were cultured in DMEM supplemented with 10% fetal bovine serum (CellMax) and 1% penicillin-streptomycin (Boster, China) at 37°C in a humidified 5% CO_2_ incubator. Empty pcDNA3.1-3xFlag vector, wild-type AGL or mutated AGL plasmid was transfected into HEK-293T cells when they were 60%–80% confluent in 12-well plates, using Lipo3000 Reagent (Invitrogen, USA) according to the manufacturer's protocols. Additionally, GFP-tagged empty vector was cotransfected as an external control of transfection.

### 2.4. Immunofluorescence Analysis

HEK-293T cells were seeded on 12-well plate slides and transfected with the wild-type or mutated AGL plasmid. 24 hours after transfection, cells were fixed with 4% paraformaldehyde (PFA) for 20 min, washed three times with PBS for 10 min each, and permeabilized with 0.5% Triton *X*-100 for 15 min. Then, the cells were incubated overnight at 4°C with primary mouse anti-Flag (1 : 100,8146, CST, USA), followed by Cy3 Goat Anti-Rabbit IgG (H + L) (1 : 100, AS007, ABclonal) fluorescent secondary antibody at 37°C for 1 h. The cell nucleus was stained with DAPI (Servicebio, China). Cells then were precisely imaged with an inverted confocal laser-scanning microscope. Finally, the images were analyzed by Image*J* software.

### 2.5. Western Blot Analysis

Cell lysates were collected 48 hours after transfection. BCA assay (Boster, China) was used to measure the protein concentration according to manufacturer's instructions. Cell lysates were separated by 8% SDS-PAGE and transferred to NC membranes (Boster, China). After 1 hour of blocking, the membrane was incubated with primary antibodies against Flag (1 : 1000, ABclonal) or GFP (1 : 10000, ABclonal) for 12 hours, continuing with secondary antibodies against IgG (1 : 5000, ABclonal) for 1.5 hours. Finally, the membranes were imaged and analyzed.

### 2.6. Measurement of Glycogen Debranching Enzyme (GDE) Activity

Cells were collected for GDE activity measurement. 48 hours after transfection, the instructions of human GDE (glycogen debranching enzyme) ELISA Kit were referred to for specific procedures. Briefly, 100 *μ*l of standards and cell samples diluted with universal diluent were added to each well of the 96 well plate precoated with human glycogen debranching enzyme (GDE) antibody and incubated for 1 h at 37°C, and then, the liquid was discarded without washing. Biotin-antibody, streptavidin-HRP working solution and TBE substrate solution were then successively added to each well; after incubating and washing, the reaction was then stopped by adding 50 *μ*l of stop solution. Finally, the absorbance (OD) was measured with a microplate reader at the wavelength of 450 nm, and the sample concentration was calculated.

### 2.7. Measurement of the Glycogen Content in HEK-293T Cell

After transfection for 48 hours, cells were harvested and subjected to intracellular glycogen analysis using a glycogen detection kit (BC0345, Solarbio), following the manufacturer's instructions. Approximately (5–10)*∗*10^6^cells were collected in centrifuge tubes, and 0.75 mL of glycogen extract was subsequently added to the cells lysed through the use of ultrasonic waves. Then, the lysate underwent a 20-minute boiling process with intermittent agitation every 5 minutes, ensuring complete lysis. After cooling, 4.25 mL of distilled water was added, and the mixture was shaken followed by centrifugation at 8000 *g* for 10 minutes at room temperature. The supernatant was collected for further experimentation. In each designated blank (*A*1), standard (*A*2), and measurement tube (*A*3), 60 *μ*L of distilled water, reagent 1, and the supernatant were added, respectively. Then, 240 *μ*L of reagent 2 was added to each well, and the mixture was homogenized by shaking. After boiling in a water bath for 10 minutes and subsequent cooling, 200 *μ*L of the mixture was dispensed into each well of a 96-well plate with triplicates. The OD value of each well was measured using a microplate reader at a wavelength of 620 nm. Finally, the intracellular glycogen content was calculated using the following formula: glycogen (mg/10^4^ cells)=0.45 *∗* (A3 − A1)/(A2 − A1)/cell count.

### 2.8. Statistical Analysis

All data are expressed as mean ± standard deviation (SD). The statistical software GraphPad Prism (version 9.0, San Diego, CA) was used for the statistical analyses. The *t*-test was performed to analyze the difference among the two groups. All *p* values <0.05 indicates statistically significant differences.

## 3. Results

### 3.1. General Clinical Characteristics

Patient 1, he was admitted to hospital due to hepatomegaly for over 20 days at the age of 1 year. His parents were nonconsanguineous, and both were normal. At first visit, his height data was lost, and weight was 11.5 kg (0.77 SD). He had hepatomegaly, and the liver size was 5.8 cm below the right midline of the clavicle. Laboratory data revealed abnormal liver function and hypoglycemia. The level of the serum creatinine, blood lipids, lactic acid, pyruvate, renal function, and blood gas was normal. Liver biopsy was not performed on the patient at the time of initial diagnosis. He was 15.6 years old at the last visit (see Table 1). Patient 2, she was admitted to hospital because of elevated serum liver enzyme levels for 1 month at the age of 2.58 years. The marriage of her parents was not consanguineous, and there was no reported family history of inherited metabolic disorders. Furthermore, her parents and the older brother exhibited normal phenotypic traits. At the first visit, her height was 83 cm (−2.44 SD) and weight was 13 kg (−0.01 SD). Hepatic palpation showed hepatomegaly. Laboratory data showed increased serum levels in liver enzymes, creatinine, lipids, lactic acid, pyruvate, and uric acid, and a high level of fasting blood glucose (7.43 mmol/L). Liver biopsy was performed, and the pathology indicated positive periodic acid-Schiff staining (PAS) which implied glycogen storage in hepatic cells. She was 9.58 years old at the last visit (see Table 1).

### 3.2. Genetic Analysis

**Figure 1:**
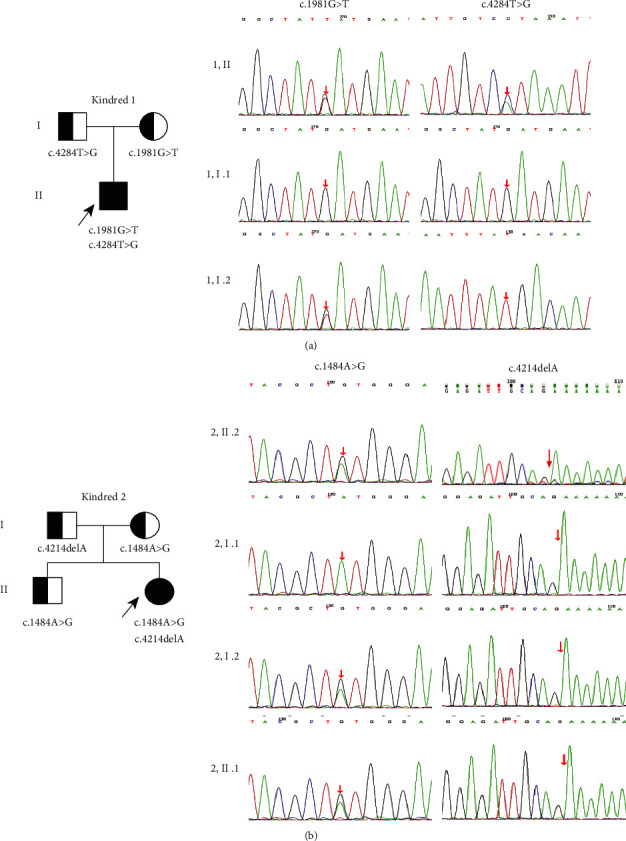
(a) Pedigree chart of patient 1 and sequencing results of the AGL mutations. (b) Pedigree chart of patient 2 and sequencing results of the AGL mutations.

**Figure 2:**
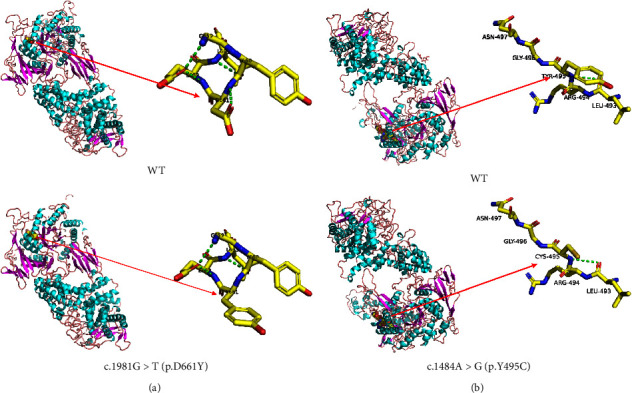
Analysis of protein conformation at two novel identified mutations in two patients with GSDIII.

Gene sequencing of patient 1 revealed two missense variants in exon15 (c.1981G > T) and exon32 (c.4284T > G), respectively, of the AGL gene (see Figure 1(a)). The c.1981G > T variant on the maternal allele causes a change of amino acid 661 from aspartic acid to tyrosine, leading to decrease in the number of hydrogen bonds, which may result in the protein instability and the configuration change (see Figure 2(a)). The c.4284T > G variant on the paternal allele has been mentioned in the database. Gene sequencing of patient 2 identified a novel missense resulting in a change from tyrosine to cysteine at position 495 (c.1484A > G (p.Y495C)) on the maternal allele and a known deletion variant c.4214delA (p. K1407Nfs∗8) on the paternal allele (see Figures 1(b) and 2(b)).

The two newly discovered variants (c.1484A > G; c.1981G > T) were forecasted to be potentially pathogenic mutations through the utilization of bioinformatics software, namely Mutation Taster, Polyphen-2, SIFT, Provean, and PyMol. Analysis of the conserved amino acids of the two mutants indicated that the site was highly conserved among different species.

### 3.3. Protein Expression and Cellular Function of Wild-Type and Mutated AGL in HEK-293T Cells

**Figure 3:**
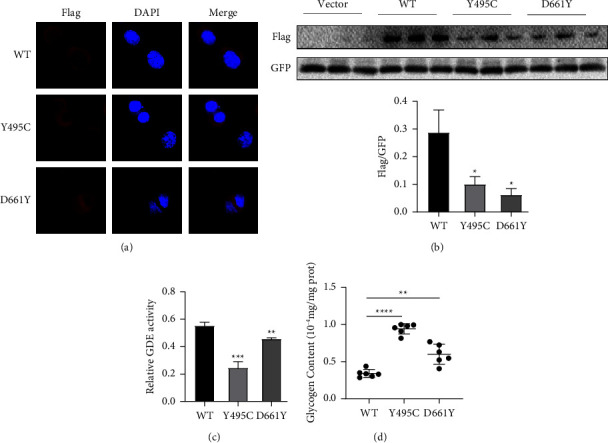
(a) Cellular location of WT and mutated AGL in HEK-293T cells; (b) expression of protein of WT and mutated AGL; (c) GDE activity of WT and mutated AGL in HEK-293T cells; (d) glycogen content of WT and mutated AGL in HEK-293T cells. Data were analyzed by GraphPad, ^*∗*^*P* < 0.05, ^*∗∗*^*P* < 0.001, ^*∗∗∗*^*P* < 0.0001.

Immunofluorescence staining showed that the location of fluorescence in cells transfected with wild-type vector was similar with the mutant ones. They were mainly located in the cytoplasm, indicating the expression site of mutant protein did not change (see Figure 3(a)). Western blot showed that the expression vectors (wild-type, c.1484A > G, c.1981G > T) were successfully transfected in the HEK-293T cells. And the expression of mutant AGLs protein similar to the flag was lower than the wild-type one (see Figure 3(b)).

To further investigate the function of the mutated AGL, we evaluated the GDE activity and glycogen content in transfected HEK-293T cells. Compared with the cells transfected with wild-type AGL, the GDE activity in the cells transfected with the variant c.1484A > G (p.Y495C) and c.1981G > T (p.D661Y) were significantly decreased, and the glycogen content one increased, which indicated the dysfunction of the two novel mutations and might be pathogenic (see Figures 3(c) and 3(d)).

### 3.4. Long-Term Follow-up Information

The two patients determined the diagnosis of GSDIII by the clinical information and the results of genetic sequencing and functional analysis. They were then treated with oral uncooked cornstarch four daily doses of 1.75 g/kg to relieve the symptom of hypoglycemia and its complications and adjusted dose according to the improvement of hypoglycemia, liver function, and weight change. Patients were followed up every three months.

Patient 1 had been treated for 14.58 years until the last visit. At the last visit, his height was 176.8 cm (1.26 SDS), and weight was 61 kg (0.82 SD). His symptom of hypoglycemia and abnormal liver function was improved remarkably, but the hepatomegaly still existed, and the liver size was 5.0 cm below the right midline of the clavicle. At the beginning, his LDH level was 564 U/L, and no significant abnormalities were found by echocardiography. After 14.58 years of treatment, his LDH dropped to 208 U/L and was within normal limits. The echocardiography results were still normal.

**Figure 4:**
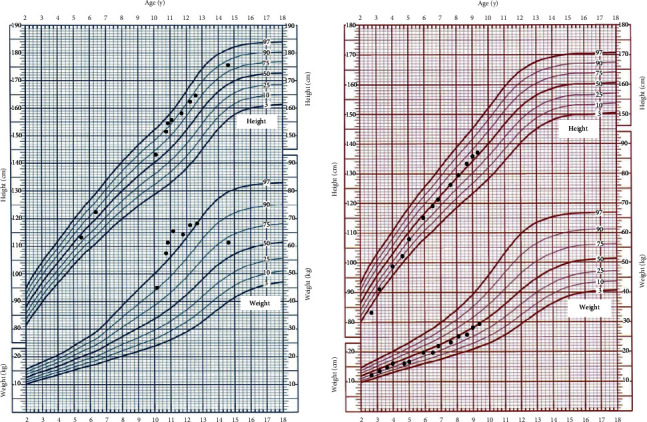
The growth curves of the two patients.

Patient 2, she had treated for 6.92 years until the last visit. At the last visit, her height was 137.1 cm (0.02 SDS), and weight was 29 kg (0.14 SD). Her symptom of hypoglycemia and abnormal liver function was improved markedly. At the beginning, her liver size was 7 cm below the right midline of the clavicle, and LDH level was 533 U/L. After a treatment period of 6.92 years, the patient's liver size reduced to 3 cm below the right midline of the clavicle, smaller than before. Additionally, her LDH levels decreased to 198 U/L, remaining within normal limits. The echocardiography results were still normal (see Figure 4).

## 4. Discussion

Glycogen storage disease type III (GSDIII) is a relatively rare autosomal recessive disorder characterized by metabolic anomalies, which was initially reported by Barbare Illingworth and Gerty Cori in 1952. [15]. The chief complaints of patients with GSDIII were usually abnormal liver function or hypoglycemic. In addition, the typical clinical manifestations include growth retardation, hyperlipidemia, and involvement of skeletal muscle and myocardium.

In this study, we identified two novel variations in the AGL gene in two unrelated Chinese families by next generation sequencing. Clinical examination of the two patients both found that the levels of liver enzymes and muscle enzymes were significantly evaluated, hepatomegaly and pathoglycemia. The two mutations (c.1484A > G; c.1981G > T) have not been reported before and were predicted to be likely-pathogenic mutations by Mutation Taster, Polyphen⁃2, SIFT, Provean, and PyMol. In subsequent functional analysis, we also discovered that the expression of AGL and the activity of GDE were mildly decreased, and the content of glycogen had a slight increase in cells transfected with mutated AGL vectors than the wild-type one which were consistent with the results of previous bioinformatics analyses. According to the criteria proposed by ACMG [16], the new missense variations c.1484A > G and c.1981G > T both could be classified as likely pathogenic (PS3 + PM2 + PP1 + PP3 + PP4).

GDE belongs to a rare bifunctional protein with two independent catalytic activities-1,4 glucan transferase and starch 1,6 glucosidase, mainly expressing in the endoplasmic reticulum(ER), which is the foundation of the variant location closely related to the clinical presentation of the patients [17]. However, GSDIII has a poor correlation between the genotype and phenotype [18, 19]. Some research has shown that the main manifestations of variation in exon 3 such as c.18_19delGA and c.16C > T were associated with GSDIIIb, and patients with IVS32-12A > G homozygosity variant have milder clinical features [13, 19]. Our study identified two newly discovered mutations (c.1484A > G; c.1981G > T) that have the potential to impair GDE activity although the severity of the enzyme activity deficiency does not appear to demonstrate a conclusive correlation with these two variant loci. The c.1981G > T mutation was found in both case 1 and his maternal parent, while case 1 exhibited a typical GSDIIIa phenotype characterized by persistent elevation in hepatic and myogenic enzyme levels since early childhood. Conversely, his mother has remained asymptomatic and without any notable irregularities. The c.1484A > G mutation occurred in the case 2, who also presented with the characteristic clinical features of GSDIIIa, associating with abnormal liver function or hepatomegaly at an early age. This mutation was also detected in case 2's mother and brother, but no discernible abnormalities have been observed thus far. Given the absence of these two mutated loci in other cases, additional investigation and validation are necessary to clarify their association with the observed phenotype.

Currently, there is no specific treatment for patients with GSDIII, and the most common approach is oral uncooked cornstarch, high fat, high protein, and other dietary management to reduce the likelihood of hypoglycemia and related complications occurring, which is helpful to stabilize the patients' conditions [1, 2, 20, 21]. However, no specific therapy has been found to be effective for patients with GSDIII. Previously, Lim et al. [22] proposed a novel gene therapy approach GSDIII using an AAV Vector Encoding a Bacterial Glycogen Debranching Enzyme, which was carried out in a mouse model and achieved good therapeutic effect. Besides, low doses of rapamycin, a specific inhibitor of mTOR, can reduce glycogen content in skeletal muscle and liver in GSDIII-affected curly models by suppressing glycogen synthase and glucose transporter 1 expression [23]. Unfortunately, these preliminary results have been obtained in animal experiments, and further exploration is needed to determine whether they can be applied to clinical practice.

## 5. Conclusion

In conclusion, we report two patients in two unrelated Chinese families with two novel variants in the AGL gene. We analyze the pathogenicity of the two novel mutations by using bioinformatics techniques and cellular functional experiments, which both indicate that the two novel mutations are pathogenic mutations with mildly increased in glycogen content. This not only expands the AGL gene variation pool but also helps us to have a further understanding of GSDIII.

## Figures and Tables

**Table 1:** Clinical information and laboratory data of the patients at the first visit.

Patients	1	2

Sex	*M*	*F*
Age of onset (years)	1	2.5
Age of diagnosis (years)	1.08	2.58
Height at diagnosis (cm)	—	83 (−2.44 SDS)
Weight at diagnosis (kg)	11.5	13
Disease course (years)	14.58	6.92
AST (U/L)	**755**	**558**
ALT (U/L)	**558**	**415**
TC (mmol/L)	2.85	4.07
TG (mmol/L)	1.48	**4.27**
HDL (mmol/L)	0.56	**0.52**
LDL (mmol/L)	1.55	2.75
LDH (U/L)	564	**533**
CK (U/L)	—	69
Creatinine (*μ*mol/L)	22	20
BUN	2.38	2.61
Fasting blood glucose (mmol/L)	**2**	**7.43**
Lactic acid (mmol/L)	1.57	**9.68**
Pyruvate (*μ*mol/L)	1.76	**142.7**
Blood ammonia	68.3	102
Uric acid (*μ*mol/L)	469.1	**341.1**
PH value	4.797	7.32
BE	−4.8	−9.5
Liver size (cm) below the right midline of the clavicle	**5.8**	**7**
PAS	—	**(+)**

AST, aspartate aminotransferase; ALT, alanine aminotransferase; TC, total cholesterol; TG, triglyceride; HDL, high-density lipoprotein; LDL, low-density lipoprotein; LDH, lactate dehydrogenase; CK, creatine kinase; BUN, blood urea nitrogen; BE, base excess; PAS, periodic acid-Schiff.

## Data Availability

The data supporting the current study are available from the corresponding author upon request.

## References

[1] Kishnani P. S., Austin S. L., Arn P. (2010). Glycogen storage disease type III diagnosis and management guidelines. *Genetics in Medicine*.

[2] Sentner C. P., Hoogeveen I. J., Weinstein D. A. (2016). Glycogen storage disease type III: diagnosis, genotype, management, clinical course and outcome. *Journal of Inherited Metabolic Disease*.

[3] Mogahed E. A., Girgis M. Y., Sobhy R., Elhabashy H., Abdelaziz O. M., El-Karaksy H. (2015). Skeletal and cardiac muscle involvement in children with glycogen storage disease type III. *European Journal of Pediatrics*.

[4] Bernier A. V., Sentner C. P., Correia C. E. (2008). Hyperlipidemia in glycogen storage disease type III: effect of age and metabolic control. *Journal of Inherited Metabolic Disease*.

[5] Michaud M., Dillier C., Selton M., Michot N., Metzdorf A., Debouverie M. (2022). Cognitive impairment in glycogen storage disease type III with severe heart failure: a case report. *Revue Neurologique*.

[6] Quackenbush D., Devito J., Garibaldi L., Buryk M. (2018). Late presentation of glycogen storage disease types Ia and III in children with short stature and hepatomegaly. *Journal of Pediatric Endocrinology & Metabolism*.

[7] Michon C.-C., Gargiulo M., Hahn-Barma V. (2015). Cognitive profile of patients with glycogen storage disease type III: a clinical description of seven cases. *Journal of Inherited Metabolic Disease*.

[8] Ponzi E., Alesi V., Lepri F. R. (2019). Uniparental isodisomy of chromosome 1 results in glycogen storage disease type III with profound growth retardation. *Mol Genet Genomic Med*.

[9] Lam C.-W., Lee A. T.-C., Lam Y.-Y. (2004). DNA-based subtyping of glycogen storage disease type III: mutation and haplotype analysis of the AGL gene in Chinese. *Molecular Genetics and Metabolism*.

[10] Beyzaei Z., Geramizadeh B., Karimzadeh S. (2020). Diagnosis of hepatic glycogen storage disease patients with overlapping clinical symptoms by massively parallel sequencing: a systematic review of literature. *Orphanet Journal of Rare Diseases*.

[11] Berling É., Laforêt P., Wahbi K. (2021). Narrative review of glycogen storage disorder type III with a focus on neuromuscular, cardiac and therapeutic aspects. *Journal of Inherited Metabolic Disease*.

[12] Lu C., Qiu Z., Sun M., Wang W., Wei M., Zhang X. (2016). Spectrum of AGL mutations in Chinese patients with glycogen storage disease type III: identification of 31 novel mutations. *Journal of Human Genetics*.

[13] Mili A., Ben Charfeddine I., Mamaï O. (2012). Molecular and biochemical characterization of Tunisian patients with glycogen storage disease type III. *Journal of Human Genetics*.

[14] Du C., Wei H., Zhang M. (2020). Genetic analysis and long-term treatment monitoring of 11 children with glycogen storage disease type IIIa. *Journal of Pediatric Endocrinology & Metabolism*.

[15] Illingworth B., Cori G. T. (1952). Structure of glycogens and amylopectins. *Journal of Biological Chemistry*.

[16] Richards S., Aziz N., Bale S. (2015). Standards and guidelines for the interpretation of sequence variants: a joint consensus recommendation of the American College of medical genetics and genomics and the association for molecular pathology. *Genetics in Medicine*.

[17] Shen J. J., Chen Y. T. (2002). Molecular characterization of glycogen storage disease type III. *Current Molecular Medicine*.

[18] Wang J., Yu Y., Cai C. (2022). The biallelic novel pathogenic variants in AGL gene in a Chinese patient with glycogen storage disease type III. *BMC Pediatrics*.

[19] Wright T. L. F., Umaña L. A., Ramirez C. M. (2022). Update on glycogen storage disease: primary hepatic involvement. *Current Opinion in Pediatrics*.

[20] Kishnani P. S., Sun B., Koeberl D. D. (2019). Gene therapy for glycogen storage diseases. *Human Molecular Genetics*.

[21] Olgac A., İnci A., Okur İ. (2020). Beneficial effects of modified atkins diet in glycogen storage disease type IIIa. *Annals of Nutrition & Metabolism*.

[22] Lim J.-A., Choi S. J., Gao F., Kishnani P. S., Sun B. (2020). A novel gene therapy approach for GSD III using an AAV vector encoding a bacterial glycogen debranching enzyme. *Molecular Therapy - Methods & Clinical Development*.

[23] Yi H., Brooks E. D., Thurberg B. L., Fyfe J. C., Kishnani P. S., Sun B. (2014). Correction of glycogen storage disease type III with rapamycin in a canine model. *Journal of Molecular Medicine*.

